# Highly Selective and Fast Response/Recovery Cataluminescence Sensor Based on SnO_2_ for H_2_S Detection

**DOI:** 10.3390/molecules28207143

**Published:** 2023-10-18

**Authors:** Bin Fan, Jing-Ru Zhang, Jia-Liang Chen, Ze-Tao Yang, Bin Li, Lin Wang, Mai Ye, Lu-Lu Zhang

**Affiliations:** Guangdong Provincial Academy of Environmental Science, Guangzhou 510045, China; zhangjrgucas@163.com (J.-R.Z.); chenjialiang_email@163.com (J.-L.C.); 15817065385@163.com (Z.-T.Y.); lishite1988@163.com (B.L.); diarmait15@163.com (L.W.); vein1980@163.com (M.Y.)

**Keywords:** SnO_2_, cataluminescence, hydrogen sulfide, sensor

## Abstract

In the present work, three kinds of nanosized SnO_2_ samples were successfully synthesized via a hydrothermal method with subsequent calcination at temperatures of 500 °C, 600 °C, and 700 °C. The morphology and structure of the as-prepared samples were characterized using X-ray diffraction, transmission electron microscopy, selected area electron diffraction, Brunauer–Emmett–Teller analysis, and X-ray photoelectron spectroscopy. The results clearly indicated that the SnO_2_ sample calcined at 600 °C had a higher amount of chemisorbed oxygen than the SnO_2_ samples calcined at 500 °C and 700 °C. Gas sensing investigations revealed that the cataluminescence (CTL) sensors based on the three SnO_2_ samples all exhibited high selectivity toward H_2_S, but the sensor based on SnO_2_−600 °C exhibited the highest response under the same conditions. At an operating temperature of 210 °C, the SnO_2_−600 °C sensor showed a good linear response to H_2_S in the concentration range of 20–420 ppm, with a detection limit of 8 ppm. The response and recovery times were 3.5 s/1.5 s for H_2_S gas within the linear range. The study on the sensing mechanism indicated that H_2_S was oxidized into excited states of SO_2_ by chemisorbed oxygen on the SnO_2_ surface, which was mainly responsible for CTL emission. The chemisorbed oxygen played an important role in the oxidation of H_2_S, and, as such, the reason for the SnO_2_−600 °C sensor showing the highest response could be ascribed to the highest amount of chemisorbed oxygen on its surface. The proposed SnO_2_-based gas sensor has great potential for the rapid monitoring of H_2_S.

## 1. Introduction

Hydrogen sulfide (H_2_S) is a noxious gas that smells like rotten eggs [1,2,3]. Even exposure to a low concentration of H_2_S can cause serious harmful effects, including damage to the respiratory and central nervous systems [4,5]. Moreover, death occurs immediately upon exposure to a high concentration of H_2_S. The threshold limit for an 8 h exposure to H_2_S is 10 ppm, which was established by the Health and Safety Executive [6]. In addition, H_2_S is also a flammable compound that can cause fires and explosions when exposed to a flame or high temperatures. However, what is more worrying is that H_2_S is found both in the natural environment and in a variety of production processes, such as oil extraction and metal smelting. In other words, H_2_S is present in the human living environment. Therefore, in order to prevent harm from H_2_S, there is an urgent need to develop high-performance gas sensors for the timely detection of H_2_S.

Cataluminescence (CTL) a kind of in situ chemiluminescence, which occurs through catalytic oxidation reactions at gas–solid interfaces [7,8]. On account of its numerous advantages, such as a rapid response, extraordinary sensitivity, high selectivity, good repeatability, and convenience, CTL-based sensors show promising application in environmental monitoring [9,10], material characterization [11,12], food analysis [13,14], catalyst evaluation [15,16], and clinical diagnosis [17,18,19]. CTL-based sensors are considered one of the most attractive and effective tools for gas sensing. In 2004, Zhang et al. pioneered the development of a CTL-based sensor for H_2_S using Fe_2_O_3_ as the sensing material [20]. Since then, the development of CTL-based sensors for H_2_S has attracted extensive attention. In particular, the Lv group has conducted a series of innovative studies on the development of a high-performance CTL-based sensor for H_2_S. Examples include a H_2_S sensor based on a hierarchical hollow microsphere and flower-like In_2_O_3_ [21], a H_2_S sensor using an α-Fe_2_O_3_/g-C_3_N_4_ composite [22], and an F-doped cage-like SiC as a metal-free sensing material for H_2_S [23]. Although significant progress has been made, there are still some drawbacks, such as the high operating temperature, poor selectivity, and complicated procedures for synthesis of the sensing material [24]. Therefore, persistent efforts should be made to overcome these drawbacks.

The sensing material directly determines the sensing performances, including the sensitivity, selectivity, reproducibility, etc. Metal oxide semiconductors have been widely developed as sensing materials owing to their high sensitivity, low cost, good reliability, and fast response [25,26]. Tin dioxide (SnO_2_) is a stable n-type wide-bandgap semiconducting metal oxide with stable chemical transduction properties [27]. SnO_2_ easily adsorbs oxygen on its surface because of its natural non-stoichiometry, providing it with high reactivity toward reducing gases [28]. Nowadays, SnO_2_ is widely used as a base material to design electrical gas sensors for the detection of harmful gas, including H_2_S [29,30,31,32]. However, electrical gas sensors based on nanostructured SnO_2_ usually have poor selectivity. It was reported that CTL produced based on a solid catalyst must meet three conditions [33]. First, the reaction must release sufficient energy, but not all of the reactions can release enough energy. Second, the reaction pathway must favor a change in the energy to form an electronically excited molecule. Third, the electronically excited molecule must release its energies via radiative transition but not nonradiative transition. Unfortunately, radiative transition may appear in low proportions. Therefore, CTL sensors show good selectivity due to the three essential conditions described above. However, a CTL sensor using SnO_2_ for H_2_S has not been reported, which inspired us to employ SnO_2_ to design a CTL sensor for H_2_S to address some of the issues in H_2_S sensing.

Herein, three kinds of SnO_2_ were synthesized by a simple hydrothermal method at three different calcination temperatures. Among them, the SnO_2_ samples annealed at 600 °C (SnO_2_−600 °C) showed the highest CTL response toward H_2_S and exhibited sufficient selectivity. Subsequently, the influencing parameters and sensing performance of the SnO_2_−600 °C sensor were investigated in detail. The sensing mechanism was also explored. Compared with most reported H_2_S sensors, the outstanding advantage of the present sensor is that it shows high selectivity at a relatively low operating temperature.

## 2. Results

### 2.1. Structural and Morphological Characterizations

**Figure 1:**
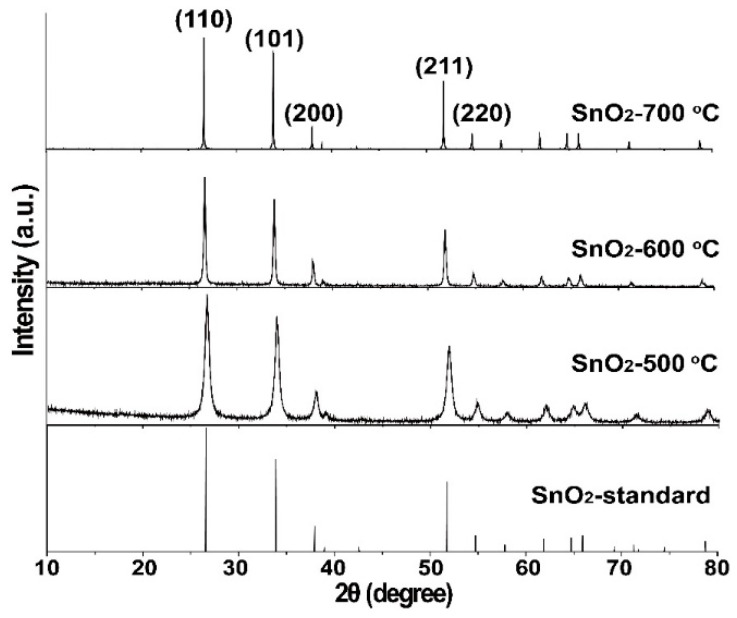
XRD patterns of the as-prepared samples.

Figure 1 shows the XRD patterns of the three samples annealed at different temperatures. Diffraction peaks at 2*θ* = 26.58°, 33.88°, 37.94°, 51.78°, and 54.76° correspond to the (110), (101), (200), (211), and (220) planes of tetragonal SnO_2_ with standard lattice constants of *a* = *b* = 4.7382 Å and *c* = 3.1871 Å (JCPDS card no. 41–1445). An increase in the calcination temperature resulted in a decrease in the full width at half maxima, which mainly results from increased crystallinity. This result agrees with those of previous reports [34,35]. In addition, no impurity peak is presented in the XRD patterns, which clearly proves that highly purified SnO_2_ was successfully synthesized.

**Figure 2:**
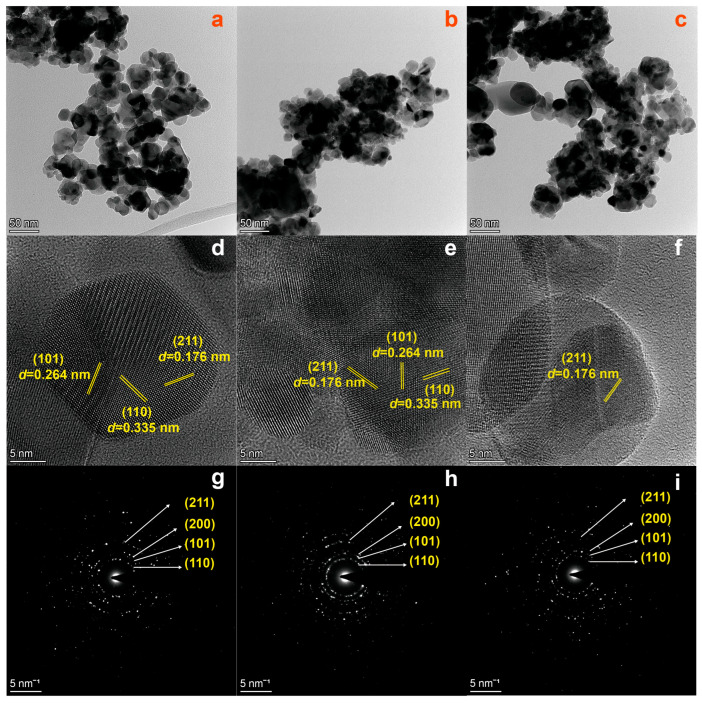
TEM images of (**a**) SnO_2_−500 °C; (**b**) SnO_2_−600 °C; (**c**) SnO_2_−700 °C. HRTEM images of (**d**) SnO_2_−500 °C; (**e**) SnO_2_−600 °C; (**f**) SnO_2_−700 °C. SAED images of (**g**) SnO_2_−500 °C; (**h**) SnO_2_−600 °C; (**i**) SnO_2_−700 °C.

The morphology and size of the as-prepared SnO_2_ samples were investigated by transmission electron microscopy (TEM) and high-resolution transmission electron microscopy (HRTEM). Figure 2a–c reveal that the three SnO_2_ samples are monodispersed nanoparticles with an average particle diameter of about 20 nm. The HRTEM images in Figure 2d,e show that both the SnO_2_−500 °C and SnO_2_−600 °C have a (101) plane with a lattice space of 0.264 nm, a (110) plane with a lattice space of 0.335 nm, and a (211) plane with a lattice space of 0.176 nm. Figure 2f shows that SnO_2_−700 °C has a (211) plane with a lattice space of 0.176 nm. The corresponding selected area electron diffraction (SAED) images in Figure 2g–i display the observed diffraction patterns consisting of concentric rings, which implies that the SnO_2_ samples have a polycrystalline structure. Meanwhile, the SAED fringe patterns are consistent with the peaks observed in the XRD spectra.

**Figure 3:**
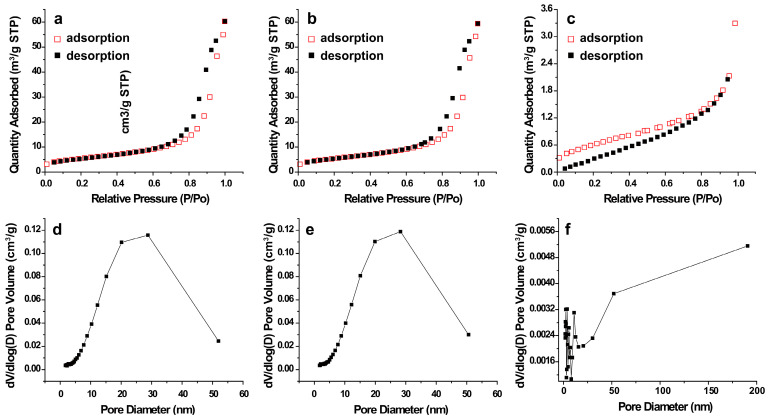
Nitrogen adsorption–desorption isotherms of (**a**) SnO_2_−500 °C; (**b**) SnO_2_−60 °C; (**c**) SnO_2_−700 °C. Pore size distribution of (**d**) SnO_2_−500 °C; (**e**) SnO_2_−600 °C; (**f**) SnO_2_−700 °C.

The specific surface areas and pore size distribution of the as-prepared samples were measured by nitrogen adsorption−desorption and obtained using Barrett−Joyner−Halenda (BJH) calculations of the adsorption branches. Figure 3a–c show that all the SnO_2_ samples produced typical type IV curves and have mesoporous features. As shown in Table 1, the BET surface areas of SnO_2_−500 °C, SnO_2_−600 °C, and SnO_2_−700 °C were 19.70, 19.73, and 2.31 m^2^/g, respectively. The Langmuir surface areas were 289.07, 295.61, and 14.00 m^2^/g, respectively, following the order of SnO_2_−600 °C > SnO_2_−500 °C > SnO_2_−700 °C. The average pore sizes of SnO_2_−500 °C and SnO_2_−600 °C were 17.41 nm and 17.02 nm, respectively, while the average pore size of SnO_2_−700 °C was not calculated because of its minuscule surface area. Obviously, SnO_2_−700 °C possessed the smallest surface area, while SnO_2_−500 °C and SnO_2_−600 °C had similar surface areas and smaller pore sizes.

**Figure 4:**
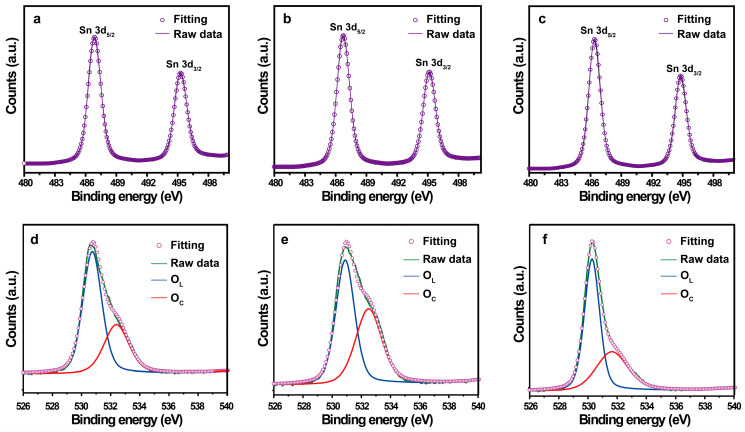
Sn 3d core level XPS spectra of (**a**) SnO_2_−500 °C; (**b**) SnO_2_−600 °C; (**c**) SnO_2_−700 °C. O 1s core level XPS spectra of (**d**) SnO_2_−500 °C; (**e**) SnO_2_−600 °C; (**f**) SnO_2_−700 °C.

The elemental and valence state analyses were performed based on the XPS spectra. Figure 4 shows the XPS spectra of SnO_2_−500 °C, SnO_2_−600 °C, and SnO_2_−700 °C, which were composed of tin and oxygen core levels. The Sn 3d peak was deconvoluted into two peaks: Sn_5/2_ and Sn_3/2_. For SnO_2_−500 °C, the Sn 3d peaks centered at the binding energies of 486.88 eV and 495.28 eV, respectively, yielding a peak-to-peak separation of 8.4 eV (Figure 4a). Similarly, SnO_2_−600 °C exhibited corresponding peaks at 486.74 eV and 495.14 eV, with a separation of 8.4 eV (Figure 4b), and SnO_2_−700 °C exhibited corresponding peaks at 486.39 eV and 494.79 eV, with the same separation of 8.4 eV (Figure 4c). The above results indicates that all the Sn 3d peaks can be assigned to the highest oxidation state of Sn^4+^ for SnO_2_ [36,37].

The measured O 1s core level spectra of the SnO_2_ samples are presented in Figure 4d–f. The asymmetric peak of O 1s was fitted by two deconvoluted peaks for all samples. The main components of the O 1s signal, centered at 530.32–530.89 eV, are attributed to the lattice oxygen (O_L_) as O^2−^ in SnO_2_. The higher binding energy peaks, centered at 532.11–532.504 eV, can be ascribed to the chemisorbed oxygen (O_c_) on the SnO_2_ surface [36,38]. The binding energy value and the peak area ratio of oxygen species in each sample are listed in Table 2. The percentages of O_C_ were 34.12%, 45.30%, and 26.97% for SnO_2_−500 °C, SnO_2_−600 °C, and SnO_2_−700 °C, respectively. The SnO_2_−600 °C sample had the highest amount of chemisorbed oxygen. Many studies have demonstrated that abundant chemisorbed oxygen can improve the sensing sensitivity.

### 2.2. Evaluation of the Sensing Materials

**Figure 5:**
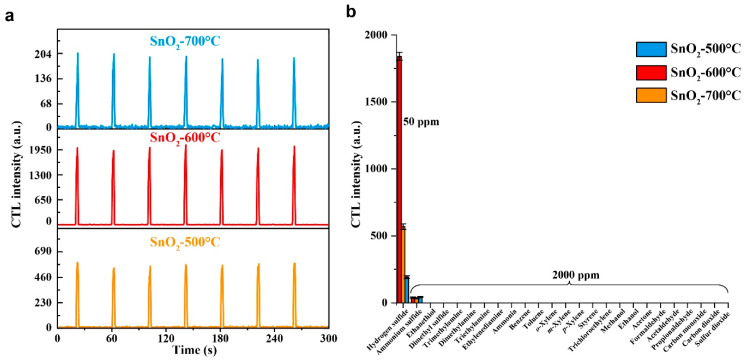
(**a**) The response curves of the three sensors toward H_2_S. (**b**) The responses of the three sensors toward 50 ppm of H_2_S and 2000 ppm of potentially interfering gases.

The three as-prepared SnO_2_ samples were used to fabricate gas sensors to sense H_2_S. Figure 5a shows the seven parallel determinations of H_2_S at 50 ppm by the three SnO_2_ sensors. The relative standard deviations (RSDs) of the response signals of the three sensors were 2.6%, 2.5%, and 3.6%, respectively, indicating that all the sensors have good reproducibility. However, the response signal of SnO_2_−600 °C was significantly higher than that of SnO_2_−500 °C and SnO_2_−700 °C. Then, the selectivity of the sensors toward H_2_S was studied by three parallel determinations of different gases at 2000 ppm, including ammonium sulfide, ethanethiol, dimethyl sulfide, trimethylamine, dimethylamine, triethylamine, ethylenediamine, ammonia, benzene, toluene, *o*-xylene, *m*-xylene, *p*-xylene, styrene, trichloroethylene, methanol, ethanol, acetone, formaldehyde, acetaldehyde, propionaldehyde, carbon monoxide, carbon dioxide, and sulfur dioxide. As Figure 5b shows, 2000 ppm of ammonium sulfide produced weak responses, but its concentration was much higher than that of H_2_S. It was found that ammonium sulfide could not produce responses from all the sensors when its concentration was reduced to 1000 ppm. It is worth mentioning that methanol, ethanol, acetone, and carbon monoxide are common interferents for H_2_S sensors [29,30,31,32,39]. However, the present sensors did not produce responses toward high concentrations of these gases, indicating that the as-prepared SnO_2_ samples are qualified for the selective sensing of H_2_S. Because SnO_2_−600 °C had the most sensitive response and possessed satisfactory selectivity, the sensing performance of a sensor based on SnO_2_−600 °C was studied in detail in the subsequent work.

### 2.3. Optimization of Conditions

**Figure 6:**
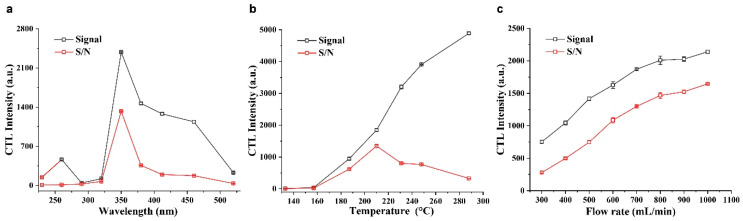
The effects of the detecting wavelength (**a**), operating temperature (**b**), and flow rate (**c**) on the CTL response signal and S/N. The concentration of H_2_S used was 50 ppm. For wavelength optimization, the operating temperature was 210 °C, and the flow rate was 800 mL/min. For temperature optimization, the detecting wavelength was 350 nm, and the flow rate was 800 mL/min. For flow rate optimization, the detecting wavelength was 350 nm, and the operating temperature was 210 °C.

The detecting wavelength, operating temperature, and flow rate have sizable effects on CTL sensing. First, the effect of the detecting wavelength on the CTL response signal and signal-to-noise ratio (S/N) was investigated by changing the optical filters in a range of 230–520 nm. As shown in Figure 6a, both the CTL response signal and the S/N value reached their maximums at 350 nm. Consequently, 350 nm was chosen as the optimal wavelength for the CTL sensing of H_2_S.

Next, the effect of the operating temperature on the CTL response signal and S/N was investigated. Figure 6b shows the change trends of the response signal and S/N versus operating temperature ranging from 133 to 288 °C. The CTL response signal increased monotonically with the operating temperature. For the S/N value, it increased with operating temperature before 210 °C and then deceased with increasing temperature, which can be mainly attributed to the background noise emitted by the thermal radiation that increases with the increasing temperature. Therefore, an operating temperature of 210 °C was chosen as the optimal working temperature, as the maximum S/N was observed at this temperature.

Finally, the effect of the flow rate on the CTL sensing of H_2_S was investigated. As Figure 6c shows, both the CTL response signal and the S/N value increased gradually with the increasing flow rate. Even when the flow rate reached the maximum rate (1000 mL/min) of the mass control flowmeter, the CTL response signal and the S/N value did not show decreasing trends. This means that the rapid oxidation rate of H_2_S on the SnO_2_−600 °C surface and the total reaction rate is controlled by the transfer rate of H_2_S from the gas phase to the SnO_2_−600 °C surface. Thus, an increase in the flow rate accelerates the total reaction rate, causing the increase in the CTL signal and S/N. Because the change in the CTL response signal and S/N tended to be stable when the flow rate exceeded 800 mL/min, a flow rate of 800 mL/min was chosen as the optimal flow rate.

### 2.4. Analytical Characteristics

**Figure 7:**
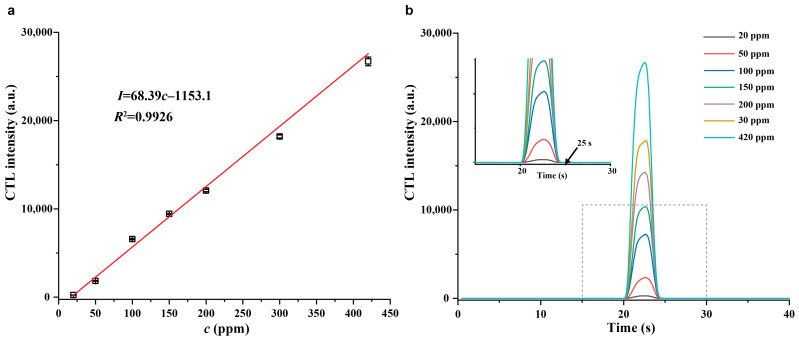
(**a**) Linear relationship between the CTL response signal and H_2_S concentration. (**b**) CTL response profiles of the sensor toward different concentrations of H_2_S. Inset: enlarged view of the CTL response profiles.

Figure 7a shows the response signal of the sensor based on SnO_2_−600 °C toward different concentrations of H_2_S under the optimal conditions. A good linear relationship between the CTL response signal and H_2_S concentration was found in the range of 20–420 ppm with a correlation coefficient of 0.9926. The linear equation was *I* = 68.39*c* − 1153.1, where the *I* is the average CTL response signal of three parallel determinations, and *c* represents the H_2_S concentration (ppm). The limit of detection (LOD) was 8 ppm at an S/N ratio of 3. The LOD of the sensor is lower than the threshold limit value of 10 ppm for H_2_S in a workplace (8 h time weighted average), indicating its promising application to monitor H_2_S in the workplace for safety management.

A comparison of the sensing performances between the present sensor and other H_2_S sensors is summarized in Table 3. The present sensor has a relatively lower operating temperature and a faster response and recovery speed than most electrical sensors. Although the electrical sensors are more sensitive than the present sensor, our sensor exhibits higher selectivity toward H_2_S. Gas sensors that can operate at low or room temperatures are highly desired for reducing the risk of gas explosions, minimizing energy consumption, and increasing security. Further endeavors could be made to improve the sensitivity and reduce the operating temperature of CTL sensors based on SnO_2_ materials via doping noble metals, loading porous materials, or engineering the oxygen vacancy.

Figure 7b displays the CTL response profiles of the sensor toward different concentrations of H_2_S under the optimal conditions. The injection time was 20 s, and it can be seen that the response signal can reach the maximum value within 3.5 s, and the maximum response signal can decay back to baseline within 1.5 s for all concentrations of H_2_S, indicating a fast response/recovery behavior of the sensor and highlighting its ability to rapidly monitor H_2_S.

### 2.5. Mechanism Study

**Figure 8:**
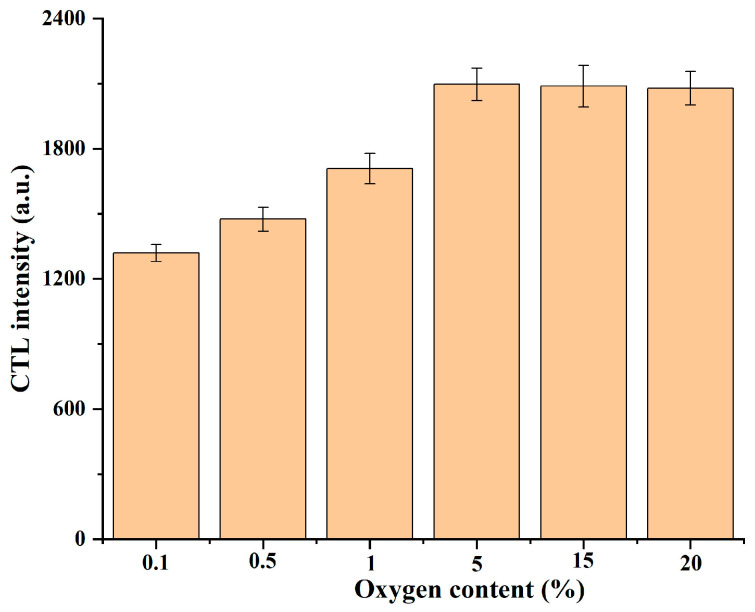
The influence of the oxygen content on the CTL intensity of the H_2_S response on the SnO_2_−600 °C surface.

SnO_2_ is a typical n-type semiconductor material; when a SnO_2_ sensor is exposed to the air atmosphere, the O_2_ molecules in the air react with SnO_2_ to produce chemisorbed oxygen via receiving electrons, such as O^2−^, O^2−^, and O^−^ [31,45]. It was reported that O^−^ is the predominant chemisorbed oxygen species on metal oxide surfaces between 100 and 300 °C [46], and thereby the O^−^ species is the preferential chemisorbed oxygen because the optimal working temperature of the sensor is 210 °C. We found that the CTL signal could not be detected on a heated ceramic heater without sintering nanosized SnO_2_, indicating that the catalyst is essential to the CTL emission. In addition, no CTL signal was detected when the air carrier was replaced by nitrogen, helium, or argon, implying that the surface chemisorbed oxygen but not the lattice oxygen takes part in the reaction. The influence of the oxygen content on CTL intensity was also investigated. As shown in Figure 8, the CTL intensity increased gradually with increasing oxygen content, and then remained stable when the oxygen content exceeded 5%. Oxygen is the second most abundant gas in the air at about 21%, indicating that the use of air as a carrier gas can provide sufficient oxygen for the oxidation of H_2_S on SnO_2_ surfaces.

In order to further explore the sensing mechanism, gas chromatography–mass spectrometer was used to identify the reaction product from the oxidation of H_2_S on the SnO_2_ surface. SO_2_ and H_2_O were detected in the exhaust gas from the oxidation of H_2_S on the SnO_2_ surface. According to the accepted theory, the generation of excited state intermediates is necessary for the generation of CTL during the reaction process [47]. It was reported that SO_2_ is a strong chemiluminescence continuum band between 250 and 500 nm with a peak wavelength of around 350 nm [48,49]. The CTL emission spectrum shown in Figure 6a also exhibits similar characteristics. Therefore, the excited state of SO_2_ (SO_2_*) is the possible luminophore, which is responsible for CTL emission from the oxidation of H_2_S on the SnO_2_ surface. According to the above discussion, the possible oxidation reaction for H_2_S on the SnO_2_ surface in air can be described as follows:

(1) $${\;O}_{2}\underset{{\mathrm{SnO}}_{2}\to }{\to }O^{-},$$

(2) $$H_{2}S+O^{-}\underset{{\mathrm{SnO}}_{2}\to }{\to }{\mathrm{SO}}_{2}^{*}+H_{2}O,$$

(3) $${\mathrm{SO}}_{2}^{*}\to {\mathrm{SO}}_{2}+\mathrm{hv}(\lambda_{\max}=350\;\mathrm{nm}).$$

The above reactions display the important role of chemisorbed oxygen in CTL emission. Abundant chemisorbed oxygen can promote the oxidation of H_2_S, enhancing the CTL intensity. The XPS results show that the order of the amount of chemisorbed oxygen is SnO_2_−600 °C > SnO_2_−500 °C > SnO_2_−700 °C, which is in accordance with the order of the sensitivity. Therefore, the amount of chemisorbed oxygen can explain the different sensitivities of the sensors based on the different SnO_2_ samples. However, extensive work is still needed to explain the underlying sensing mechanism. For example, the adsorption behavior of H_2_S and the evolution of oxygen species on heated SnO_2_ surfaces should be studies by in situ diffuse reflectance infrared Fourier transform spectroscopy or in situ Raman spectroscopy.

## 3. Experimental Section

### 3.1. Synthesis of SnO_2_

Approximately 7.012 g (0.02 mol) of SnCL_4_·5H_2_O was dissolved in 80 mL deionized water and stirred for 15 min for complete dispersion; the final concentration of SnCL_4_·5H_2_O was 0.25 mol/L. Then, 10 mL of 14.8 mol/L ammonium hydroxide (28% NH_3_ in H_2_O) was added dropwise into the solution, and the mixture was stirred continuously for 15 min. After that, the obtained solution was transferred into a Teflon-lined stainless steel autoclave and kept at 100 °C for 12 h. After naturally cooling down to room temperature, the as-synthesized precursors were washed several times with deionized water and ethanol and dried at 60 °C for 12 h in an oven. Then, the as-prepared powders were annealed at 500 °C, 600 °C, and 700 °C for 3 h, and the resulting annealed SnO_2_ samples were labeled as SnO_2_−500 °C, SnO_2_−600 °C, and SnO_2_−700 °C, respectively.

### 3.2. Characterization and Apparatus

The phase structures of the SnO_2_ samples were investigated using powder X-ray diffraction (XRD, Rigaku, Ultima IV) with Cu Kα radiation (λ = 1.5406 Å). The morphology and exposed surfaces of the SnO_2_ samples were studied by transmission electron microscopy (TEM; Titan G260-300 FEI, Stanford, CA, USA). The specific surface areas and pore size distribution of the SnO_2_ samples were investigated using a surface area analyzer (ASAP2460, Micromeritics, Norcross, GA, USA). The chemical status and the composition of elements of the SnO_2_ samples were investigated by K-Alpha X-ray photoelectron spectroscopy (XPS; Thermo Scientific, Waltham, MA, USA) with monochromatic Al Kα X-ray radiation.

### 3.3. Fabrication of the Gas Sensor

**Figure 9:**
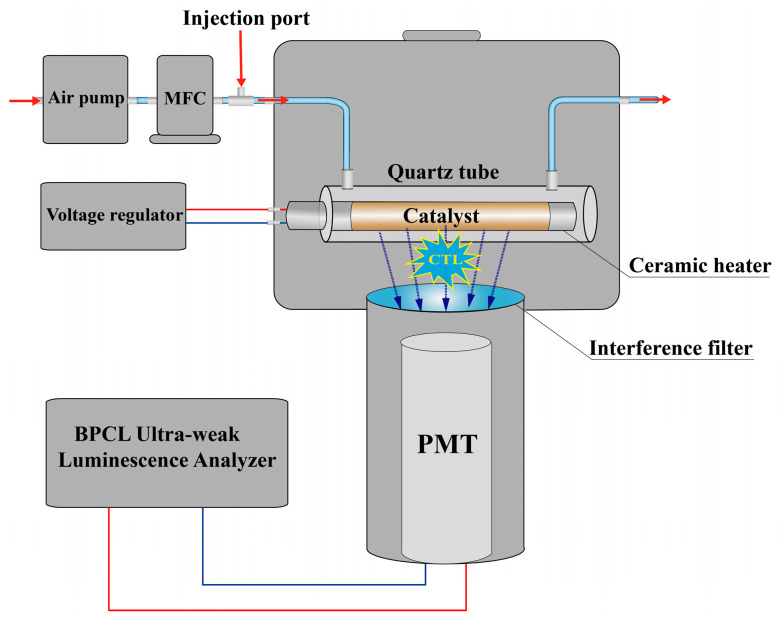
The schematic diagram of the sensing setup.

The detailed fabrication methods of the SnO_2_ sensors were similar to those reported in previous works [8,10], and a schematic diagram of the sensing setup is shown in Figure 9. Briefly, the as-prepared SnO_2_ samples were mixed with deionized water to obtain a suspension. The suspension was then brush-coated on the surface of a ceramic heater (35 W, diameter is 0.5 cm) to obtain a thin layer (about 1 mm in thickness). The ceramic heater was placed in a homemade quartz tube (with a length of 9 cm and an inner diameter of 1.2 cm) with a gas inlet and outlet. The quartz tube was placed into the sample chamber of a commercial BPCL ultra-weak luminescence analyzer (Guangzhou Microphotonics Technologies Co., Ltd., Guangzhou, China) equipped with a photomultiplier (tube type: GDB-52) at the bottom of sample chamber. The sensors were aged at 400 °C for 20 min in a stable airflow at 800 mL/min. An air pump was used to carry and transport the sample, and a mass flow controller was used to control the flow rate. A voltage regulator was used to adjust the operating temperature. The detecting wavelength was changed using different interference filters. The sample was injected into the injection port, and then the CTL signal was recorded and processed by the BPCL ultra-weak luminescence analyzer.

## 4. Conclusions

In conclusion, three kinds of nanosized SnO_2_ samples were synthesized through a hydrothermal method. Structural characterizations indicated that the order of the amount of chemisorbed oxygen of the three nanosized SnO_2_ samples was SnO_2_−600 °C > SnO_2_−500 °C > SnO_2_−700 °C. The gas sensing results indicated that the sensors based on three nanosized SnO_2_ samples all exhibited high selectivity toward H_2_S, but the SnO_2_−600 °C sensor exhibited the highest sensitivity, which can be attributed to the SnO_2_−600 °C process resulting in the highest amount of chemisorbed oxygen. The mechanism study showed that H_2_S was oxidized into SO_2_ and H_2_O by chemisorbed oxygen on the SnO_2_ surface. The excited state of the SO_2_ produced by the oxidation reaction was mainly responsible for CTL emission. The designed CTL sensor for H_2_S has good prospects for the rapid detection of H_2_S in the fields of environmental monitoring, food safety, and occupational health, owing to its advantages of sufficient sensitivity, high selectivity, and rapid response and recovery capacity.

## Figures and Tables

**Table 1:** The BET, Langmuir, and BJH results for the three samples.

Sample	BET Surface Area (m^2^/g)	Langmuir Surface Area (m^2^/g)	BJH (nm)
SnO_2_−500 °C	19.70	289.07	17.41
SnO_2_−600 °C	19.73	295.61	17.02
SnO_2_−700 °C	2.31	14.00	-

**Table 2:** XPS results of different chemical states of O elements at the surface of the as-prepared SnO_2_ nanomaterials.

Sample	Binding Energy (eV)		Percentage (%)	
	O_L_	O_C_	O_L_	O_C_
SnO_2_−500 °C	530.74	532.39	65.88	34.12
SnO_2_−600 °C	530.89	532.50	54.70	45.30
SnO_2_−700 °C	530.32	532.11	73.03	26.97

**Table 3:** Comparison of the sensing performances of H_2_S sensors.

Principle	Sensing Material	Operating Temperature (°C)	Selectivity	Res./Rec. Time (s)	Linear Range (ppm)	LOD (ppm)	Ref.
Electricity	0.1 wt% V-doped SnO_2_	350	Little response to NO (5 ppm), NO_2_ (5 ppm), SO_2_ (500 ppm), and ethanol (50 ppm).	2 s/few min	0.25–10	0.08	[29]
Electricity	SnO_2_-Al (1: 0.33)	350	Moderate response to 10 ppm of ethanol, ammonia, and toluene.	35/ND	ND	ND	[30]
Electricity	SnO_2_ nanowires	250	Moderate response to 2000 ppm of ethanol and CO and strong response to NO_2_ (3 ppm).	2.3/ND	0.2–10	ND	[31]
Electricity	NiO@SnO_2_	240	Weak response to 0.5 ppm of NH_3_, toluene, formaldehyde, and NO_2_.	37/50	0.1–50	0.0015 (in theory)	[32]
Resistance	Ni-doped ZnO nanorod	200	Moderate response to 100 ppm of methane, toluene, methanol, and ethanol.	48/60	5–40	ND	[40]
Electricity	MoO_3_ nanoflakes	300	No response to NH_3_, formaldehyde, benzene, and CO. Weak response to SO_2_ and ethanol.	ND	0.5–30	ND	[41]
Electricity	CuO/SnO_2_	200	Weak response to 200 ppm of SO_2_ (200 ppm) and 1000 ppm of methane, hydrogen, and acetylene.	ND	0.15–10	0.15	[42]
CTL	Flower-like In_2_O_3_	400	Weak response to methyl sulfide and ethanol. No response to methanol, propanol, benzene, cyclohexane, etc.	5/25	1317.6–13,176	329	[21]
CTL	α-Fe_2_O_3_/g-C_3_N_4_	183	No response to 64 mg/L of methanol, ethanol, *n*-propanol, isopropanol, *n*-butanol, isobutanol, formaldehyde, and acetaldehyde.	0.1/0.6	580–4618	329	[22]
CTL	F-doped cage-like SiC	298	Weak response to 64 ppm of 1-dodecanethiol and 1-thioglycerol. No response to 64 ppm of ethanol, isobutanol, tert-butyl alcohol, *n*-pentanol, methanol, acetone, etc.	0.1/0.6	6.1–30.4	3.0	[23]
CTL	Camellia-like NiO	246	Weak response to 65 ppm of formaldehyde, acetaldehyde, formic acid, acetic acid, acetone, ether, carbon disulfide, methyl mercaptan, ethyl mercaptan, etc.	0.2/0.4	0.8–30.8	0.3	[24]
CTL	β-MnO_2_	224	Weak response to acetone, 2-propanol, propionaldehyde, isobutanol, n-propanol, ethanol, and methanol.	0.3/0.4	1601–19,173	184	[39]
CTL	F/O-Si_3_N_4_	230	Weak response to 64 ppm of ether and acetone. No response to ethanol, isobutanol, tert-butyl alcohol, *n*-pentanol, methanol, acetone, cyclohexanone, etc.	0.5/1	322–5382	17.8	[43]
CTL	2D WS_2_ nanosheets	187	No response to methanol, ethanol, acetone, formaldehyde, acetaldehyde, formic acid, carbon disulfide, benzene, etc.	0.2/0.2	171–4203	40	[44]
CTL	SnO_2_	210	Weak response to 2000 ppm of ammonium sulfide. No response to 2000 ppm of ethanethiol, dimethyl sulfide, trimethylamine, dimethylamine, triethylamine, ethylenediamine, ammonia, benzene, toluene, *o*-xylene, *m*-xylene, *p*-xylene, styrene, trichloroethylene, methanol, ethanol, acetone, formaldehyde, acetaldehyde, propionaldehyde, CO, CO_2_, and SO_2_, and 1000 ppm of dimethyl sulfide.	3.5/1.5	20–420	8	This work

## Data Availability

The data presented in this study are available upon request from the corresponding authors.
